# GM-CSF enhanced the effect of CHOP and R-CHOP on inhibiting diffuse large B-cell lymphoma progression via influencing the macrophage polarization

**DOI:** 10.1186/s12935-021-01838-7

**Published:** 2021-03-02

**Authors:** Yu Zhang, Jingjing Xiang, Xianfu Sheng, Ni Zhu, Shu Deng, Junfa Chen, Lihong Yu, Yan Zhou, Chenjun Lin, Jianping Shen

**Affiliations:** 1 Department of Hematology, First Affiliated Hospital of Zhejiang Chinese Medical University, 54 Youdian Road, 310006 Hangzhou, China; 2 First Medical College of Zhejiang Chinese Medical University, Hangzhou, China

**Keywords:** GM-CSF, CHOP, R-CHOP, Diffuse large B-cell lymphoma, Tumor microenvironment, Macrophage

## Abstract

**Background:**

Diffuse large B-cell lymphoma (DLBCL) is a common type of the Non-Hodgkin lymphomas (NHLs) formed by the neoplastic transformation of mature B cells. As the first-line therapeutics, CHOP (cyclophosphamide/doxorubicin/vincristine/prednisone) chemotherapy and R-CHOP (Rituximab + CHOP), either using alone or in combination with GM-CSF, have achieved great efficacy in DLBCL patients. However, the underlying mechanisms are still largely unknown.

**Methods:**

In the present study, the combination use of CHOP and R-CHOP with GM-CSF was used to evaluate their effects on the tumor immune microenvironment of DLBCL. CHOP and R-CHOP administration was found to inhibit the growth and metastasis of DLBCL, with a higher efficacy in R-CHOP-challenged DLBCL mice. The anti-tumor effect of CHOP and R-CHOP was further amplified by GM-CSF.

**Results:**

CHOP and R-CHOP therapeutics potentiated the anti-tumor properties of macrophages, as evidenced by the increased M1 macrophage and the decreased M2 macrophage accumulation in DLBCL-bearing mice. In a co-culture system, macrophages primed with CHOP and R-CHOP therapeutics inhibited multiple malignant behaviors of DLCBL cells. Mechanistically, CHOP/R-CHOP suppressed the activation of AKT signaling. These anti-tumor effects of CHOP/R-CHOP were all augmented by GM-CSF.

**Conclusions:**

Our work provided new insights into the immune-regulatory roles of CHOP and R-CHOP in the treatment of DLBCL, as well as the synergistic effects of GM-CSF in CHOP and R-CHOP therapeutics. Although our results suggest the synergistic effect of GM-CSF on DLBCL already sensitive to CHOP and R-CHOP, however, future studies are warranted to explore the role of GM-CSF on R-CHOP-resistant DLBCL.

*Trial registration *Not applicable.

## Background

Diffuse large B-cell lymphoma (DLBCL) is one type of the Non-Hodgkin lymphomas (NHLs) known as an aggressive malignancy of the mature B cells [1, 2]. The standard therapeutic strategies for DLBCL at present include CHOP (cyclophosphamide/doxorubicin/vincristine/prednisone) chemotherapy or the combination rituximab and CHOP (R-CHOP) chemotherapy [3, 4]. This approach generally leads to the significant improvement in the overall survival of patients with NHLs. However, there are still more than 30 % DLBCL patients lacking of sensitivity to CHOP or R-CHOP treatment [5]. As an immunoadjuvant, granulocyte-macrophage colony-stimulating factor (GM-CSF) has been proved to be effective in tumor immunotherapy [6, 7]. Combined R-CHOP treatment with GM-CSF can further improve the prognosis of the elderly DLBCL patients [8, 9]. Nevertheless, the exact mechanisms underlying the therapeutic role of CHOP and the synergistic effect of Rituximab and GM-CSF are still elusive.

Human cancers are characterized by highly genetic heterogeneity which is caused not only by tumor cells themselves, but also by the plasticity of tumor microenvironment (TME) [10]. Increasing evidence indicates that the TME contributes significantly to B-cell lymphoma pathogenesis, progression, drug-resistance and metastasis [11]. In addition to tumor cells, the TME is composed of a mixture of mesenchymal stem cells (MSCs), immune cells (T cells, B cells, and dendritic cells, macrophages), fibroblasts, extracellular matrix, and blood vessels [12]. Among these cell populations, tumor-associated macrophages (TAM) play a predominant role in shaping TME via multiple mechanisms, including secreting cytokines/chemokines, regulating T cell infiltration/differentiation/function, or promoting angiogenesis [13]. Most macrophages originate from monocytic precursors in the bone marrow or peripheral blood. Based on their functional features, macrophages can be roughly divided into two polarization states: classically activated macrophages (M1) or alternatively activated macrophages (M2) [14]. The phenotypes and functions of TAMs are considered to resemble M2 macrophages, which generally exert pro-tumoral and pro-angiogenesis effects in the TME. In contrast, M1 macrophages mainly exert anti-tumor effect [15]. Moreover, M2 macrophages are associated with the poor clinical outcome of patients with DLBCL [16]. Repolarization of M2 macrophages into M1 phenotype is thought to inhibit tumor progression. In the present study, DLBCL mice was challenged with CHOP or R-CHOP and combination of GM-CSF, in order to investigate their different therapeutic efficacy on tumor growth and metastasis. Importantly, we put emphasis on their roles in shaping TME, especially how these therapeutic strategies impacted the frequencies of tumor-infiltrating immune cells. In addition, the co-culture of DLBCL cells with macrophages in the presence of CHOP or R-CHOP and combination of GM-CSF was used to evaluate their effects on TAMs, and TAM-mediated tumor cell behavior. Finally, the signal pathway involved in the synergistic effect of GM-CSF on CHOP for DLBCL treatment was evaluated. Although the synergistic effect of GM-CSF on DLBCL already sensitive to CHOP and R-CHOP, however, future studies are warranted to explore it’s role on R-CHOP-resistant DLBCL.

## Methods

### Cell culture and treatment

The diffuse large B-cell lymphoma cell line SU-DHL-4 was obtained from Cell Bank of Chinese Academy of Sciences. All cells were cultured in RPMI-1640 medium (Gibco, USA) supplemented with 10 % FBS (fetal bovine serum, Gibco, USA), 100 U/mL penicillin and 100 µg/mL streptomycin under 5% CO_2_ at 37℃ in a humidified atmosphere. THP-1 cells were also purchased from Cell Bank of Chinese Academy of Sciences and cultured in RPMI-1640 medium (Gibco, USA) supplemented with 10% FBS (fetal bovine serum, Gibco, USA), 10 mM Hepes (sigma, USA), 1 mM pyruvate (Sinopharm, Shanghai, China), 2.5 g/L D-glucose (sigma, USA) and 50pM β-mercaptoethanol (sigma, USA). For polarization induction, THP-1 cells were incubated with 100 ng/mL PMA (sigma, USA) for 48 h, followed by three-times washes using fresh complete RPMI-1640 medium and culturing using FBS-free RPMI-1640 medium, allowing THP-1 cells at un-activated macrophages (M0, verification by flow cytometry using CD14 and CD68 antibodies). Then cells were subjected to 20 ng/mL IL-4 (sigma, USA) and 20 ng/mL IL-13 (sigma, USA) for addition 24 h. The activated M2 macrophages were verified through flow cytometry using CD163 antibody.

### Animal

6–8 weeks male SCID mice were obtained from Charles River Co. LTD (Beijing, China). Mice were housed in specific-pathogen-free (SPF) room under controlled temperature (24 ± 0.5°C) and humidity. All mice were exposed to a 12-h light–dark cycle with free access to standard rodent chow and water. All animal studies were approved by the Animal Welfare Committee of Research Organization of Zhejiang Provincial Hospital of Chinese Medicine (2016-KL-018-02). After a week of adjustable feeding, animal tumor model was established by intravenous injection of 5 × 10^6^ SU-DHL-4 cells. Then all mice were randomly divided into five groups: model group, CHOP group, CHOP plus GM-CSF group, R-CHOP group and R-CHOP plus GM-CSF group. The induction process for CHOP group was as follows: the injection of cyclophosphamide (4.0 mg/each, sigma, US), doxorubicin (0.27 mg/each, sigma, US) and vincristine (7.5 µg/each, sigma, US) on days 1 and 22 (twice/each agent), and the injection of prednisone (0.54 mg/each) on days 1 to 5 and days 22 to 26 (ten times/each mice). For the using of GM-CSF in CHOP mice, except for the agents above used in CHOP model, 25 µg/kg GM-CSF (sigma, USA) was given subcutaneously on days 3 to 10, and days 23 to 28. In the group of R-CHOP, another drug, rituximab (2.0 mg/each, Roche, US) was injected every 3 weeks along with the injection of cyclophosphamide, doxorubicin, vincristine and prednisone. For the administration of GM-CSF in R-CHOP group, the agents for CHOP induction, GM-CSF and rituximab were subjected to mice according to the above method. On days 45 after the initial injection, blood samples were collected and all animals were euthanized by taking an overdose of carbon dioxide. Then lymph tissue, liver tissue, spleen tissue, lung tissue, kidney tissue and intestinal tissue were collected. The tumor location was photographed and analyzed.

### Hematoxylin & eosin (HE) staining

Mouse lung and kidney tissue samples contained tumor site were collected and cut into the appropriate size. The specimens were embedded using OCT embedding medium. Then 7 µm-thickness freezing slice was obtained using freezing microtome (Leica, US). The frozen sections were fixed for 20 s and washed with water for 2 s. Then the sections were subjected to hematoxylin for 60 s, and washed using running water for 10 s. After differentiation using 1% hydrochloric acid ethanol for 3 s, the sections were washed with running water and incubated with alkaline water for 20 s. After rinsing with running water, the slices were stained with eosin staining for 20 s. Then the sections were dehydrated with gradient alcohol. Subsequently, the sections were placed in dimethylbenzene for vitrification. After mounting with neutral balsam, the slices were placed in oven for 15 min at 65 ℃. Eventually, the staining results were observed by microscope.

### Immunohistochemical staining

Tumor tissues in lung and kidney tissue were fixed by formalin and embedded by paraffin. After deparaffinization in xylene and rehydration in gradient ethanol, antigen retrieval was performed by 0.01 M citrate salt solution (PH 6.0) using high pressure method. Then, the slices were cleaned by PBST and the endogenous peroxidase was neutralized by 3% H_2_O_2_ for 25 min. After blocking with 5 % BSA for 1 h at room temperature (RT), the sections were washed with PBST and incubated using primary antibody against CD20 (1:2000, abcam, Cambridge, United Kingdom) at 4°C overnight. The next day, all sections were incubated with secondary antibody labeled with streptavidin horseradish peroxidase (HRP) for 50 min at RT (1:2000, abcam). Antigen-antibody complexes were visualized after staining with DAB (ZL1-9081, ZSGB-BIO, China). Then the slices were re-stained with hematoxylin for 3 min, differentiated using 1% hydrochloric acid alcohol and incubated with ammonium hydroxide for 10 s. After dehydration using graded ethanol and vitrification by dimethylbenzene, the sections were mounted with neutral balsam. Photograph observation was performed under a Biological inverted microscope (IX51, Olympus, Japan). Comprehensive analysis included measuring staining intensity and the number of positive cells.

### ELISA

ELISA was employed to determine the changes in protein expression. In brief, equal peripheral blood was collected from mice in each group. Subsequently, blood samples were subjected to centrifuge at 4000 rpm for 30 min at 4 ℃ for serum observation. In addition, the supernatant of the cell culture medium of M0 and M2 macrophages was collected. Then serum β2-MG (Termofisher, US) and LDH (Termofisher, US), IL-12 p70 (Termofisher, US) in the supernatant fluid of M0 macrophages and IL-12 p70 and IL-10 (Termofisher, US) in the supernatant fluid of M2 macrophages were assessed using ELISA kits according to the manufactures’ instructions. The staining results were determined by multimode reader (BioTEK synergy H1) at the wavelength of 450 nm.

### Flow cytometry

The collected fresh lymphoma tissue was placed in culture dish and cut into pieces. The tissue pieces were washed with PBS and digested in 40 ml RPMI1640 medium containing 3600 u DNAse, 50 µg collagenase and 125 u hyaluronidase at 37°C for 1 h. After the termination of reaction using complete RPMI1640 medium, a homogeniser was used to break up the tissue mass. The tissue fragments were filtered with a 100 µM filter, and then the cell mass was removed with 30 µM filter after transient centrifugation. The single-cells were collected after centrifugation at 1500 rpm for 10 min and resuspended in 5ml PBS. The single cell suspensions were mixed with lymphocyte separation medium (Ficoll) and centrifugated at 2000 rpm for 20 min. The upper superstratum was the mononuclear cell suspensions. After removing the lymphocyte separation medium using centrifugation at 1500 rpm for 10 min, the mononuclear cell pellets were collected and resuspended with binding buffer. Then dendritic cells were labeled with CD11c^+^ antibody (1:200, BD, US), M1 and M2 macrophages were labeled with CD68^+^ (1:200, abcam, UK) and HLA-DR^+^ (1:200, abcam, UK), and CD68^+^ and CD163^+^ for 30 min in darkness, respectively. After resuspension using 2 ml PBS, the mixture was centrifugated at 1500 rpm for 5 min. The cell pellets were resuspended and analysed by a FACS Verse flow cytometer (BD Biosciences).

### CCK-8

Briefly, M0 and M2 macrophages were exposed to CHOP (8.32 ug/mL cyclophosphamide, 0.55 ug/mL doxorubicin, 0.016 ug/mL vincristine and 1.109 ug/mL prednisone), CHOP plus 10 ng/mL rhGM-CSF, R-CHOP (CHOP plus 10 µg/mL rituximab), and R-CHOP plus 10 ng/mL rhGM-CSF. After 6 days of induction, the macrophages (M0 and M2) were co-cultured with SU-DHL-4 cells using Transwell apparatus (Corning, US), respectively. Macrophages were seeded in the upper chamber and SU-DHL-4 cells were plated in the lower chamber. During the co-culture, the medium contained different drugs in macrophages was not removed. Then 10 µl CCK-8 solution was added into each well and incubated at 37 °C for 2 h. Finally, the optical density (OD) was read at 450 nm using a Bio-Rad iMark plate reader.

### Cell apoptosis

Macrophages (M0 and M2) were exposed to CHOP, CHOP plus rhGM-CSF, R-CHOP and R-CHOP plus rhGM-CSF for 6 days. Then SU-DHL-4 cells and macrophages were co-cultured in the Transwell apparatus for addition 24 h. SU-DHL-4 cells were collected and washed with PBS for 3 times. All cells were stained with annexin V-FITC/propidium iodide (Jianchen, Nanjing, China) following the manufacturer’s instructions. Cell apoptosis were determined using a BD FACSCalibu flow cytometry system (Becton Dickinson, NJ, USA).

### Transwell assay

In brief, macrophages (M0 and M2) were subjected to CHOP, CHOP plus rhGM-CSF, R-CHOP and R-CHOP plus rhGM-CSF for 6 days of induction. Subsequently, the macrophages were seeded in the lower chamber (24-well plate) and maintained the induction of the above agents. The lower chamber was filled with 600 µl of medium containing 10 % FBS. 1 × 10^5^ SU-DHL-4 cells were collected and resuspended with 100 µl serum free medium. SU-DHL-4 cell suspension was seeded onto the upper chamber with or without matrigel (BD biosciences, MA USA) of Transwell apparatus in 24-well plate (Corning, NY, USA) with a pore size of 8.0 µm. After incubation for 24 h at 37°C in 5 % CO_2_, the SU-DHL-4 cells migrated/invaded to the supernatant of low chamber were collected and the migration rates were evaluated by CCK8 experiment.

### Western blotting

Cells were lysed on ice by lysis buffer (Beyotime, Shanghai, China), and the total protein was extracted by centrifugation at 12,000 g for 10 min at 4℃. The mixture of equal protein and 5×loading buffer was separated by SDS-PAGE and then transferred onto an activated PVDF membrane (Boster Biological Tech co.ltd., Wuhan, China). Followed the blocking with skim milk solution for 1 h, the PVDF membrane was incubated with relative primary antibodies against PTEN (1:1000, Abcam, UK), p-PTEN (1:1500, Abcam, UK), AKT (1:1000, Abcam, UK), p-AKT (1:1000, Abcam, UK), PARP (1:1000, Abcam, UK) and cleaved-PARP (1:1000, Abcam, UK) at 4 ℃ overnight. GAPDH (1:1000, Abcam, UK) was served as the internal control. The next day, after incubating with the appropriate horseradish peroxidase-conjugated secondary antibodies (dilution, 1:2000; Beyotime Institute of Biotechnology) for 1 h at room temperature, the blots were visualized by visualized by Versa Doc (Bio-Rad Laboratories, Inc.).

### Statistical analysis

All the data are presented as the means ± SD using SPSS 21.0 software. One-way ANOVA (LSD test) was used to assess the difference between multiple groups (Graph Pad Prism 7.0). P < 0.05 was considered as statistical significance.

## Results

### GM-CSF enhanced the effect of CHOP and R-CHOP on inhibiting DLBCL

**Fig. 1:**
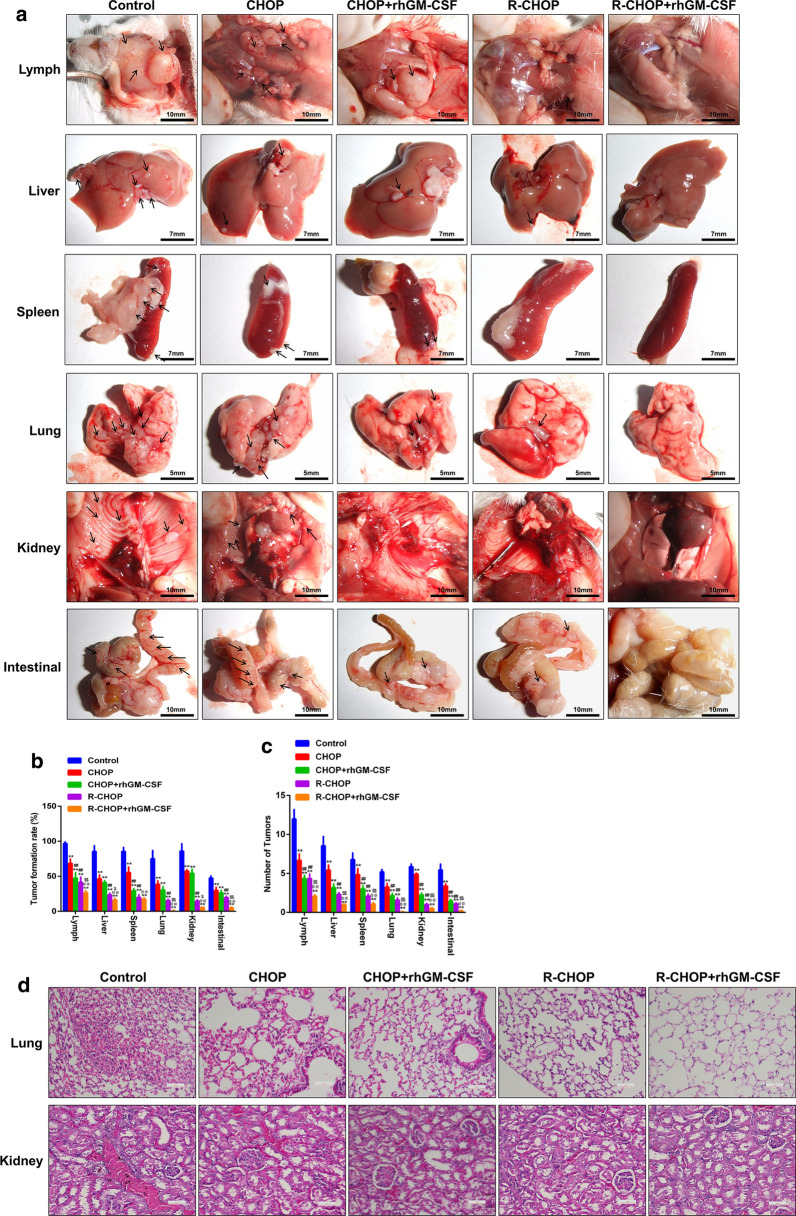
GM-CSF enhanced the effect of CHOP and R-CHOP on inhibiting DLBCL. **a** Representative pictures showing the progression of DLBCL and its metastasis to lymph, liver, spleen, lung and small intestine. **b**, **c** The tumor formation rate and the number of tumor nodules in each organ were calculated. **d** HE staining was performed to determine the pathological changes of lung and kidney lymphoma in mice of each group. **p < 0.01 vs. Control, ##p < 0.01 vs. CHOP, @@p < 0.01 vs. CHOP + GM-CSF, $p < 0.05 and $$p < 0.01 vs. R-CHOP. Data are representative of at least three independent experiments

In order to investigate the impact of different therapeutic strategies on DLBCL, SU-DHL-4 cells were inoculated i.v. into nude mice. 45 days post inoculation, mice exhibited mental malaise, emaciation, hair erection and sluggish activity. After dissection, tumor nodules were found in the lymph, liver, spleen, lung and small intestine of mice, which confirmed the successful establishment of *DLBCL model.* Next, we explored the therapeutic efficacy of CHOP or R-CHOP and combination of GM-CSF against tumor growth by measuring the number and volume of tumor nodules. As shown in Fig. 1a–c, although tumor development was also observed in multiple organs in mice receiving all of the four reagents, CHOP and R-CHOP administration significantly reduced both the tumorigenesis rate and tumor numbers, as evidenced by the fewer tumor nodules. And smaller tumor size particularly was observed in CHOP groups compared to R-CHOP groups, implying that the anti-tumor effect was more obvious in R-CHOP group than that in CHOP. On the other hand, GM-CSF also amplified the inhibitory effect of CHOP and R-CHOP on tumor progression. These findings indicated the anti-DLBCL effects of CHOP and R-CHOP, which could be further accelerated by the co-administration of GM-CSF.

In terms of pathological characteristics, a large number of tumor cells as well as inflammatory cells were observed in the lungs and kidneys in control mice. These pathological changes were reduced by CHOP and R-CHOP administration, and were further inhibited by the co-treatment with GM-CSF (Fig. 1d).

### GM-CSF enhanced the effect of CHOP and R-CHOP on the production of β2-MG, LDH and CD20

**Fig. 2:**
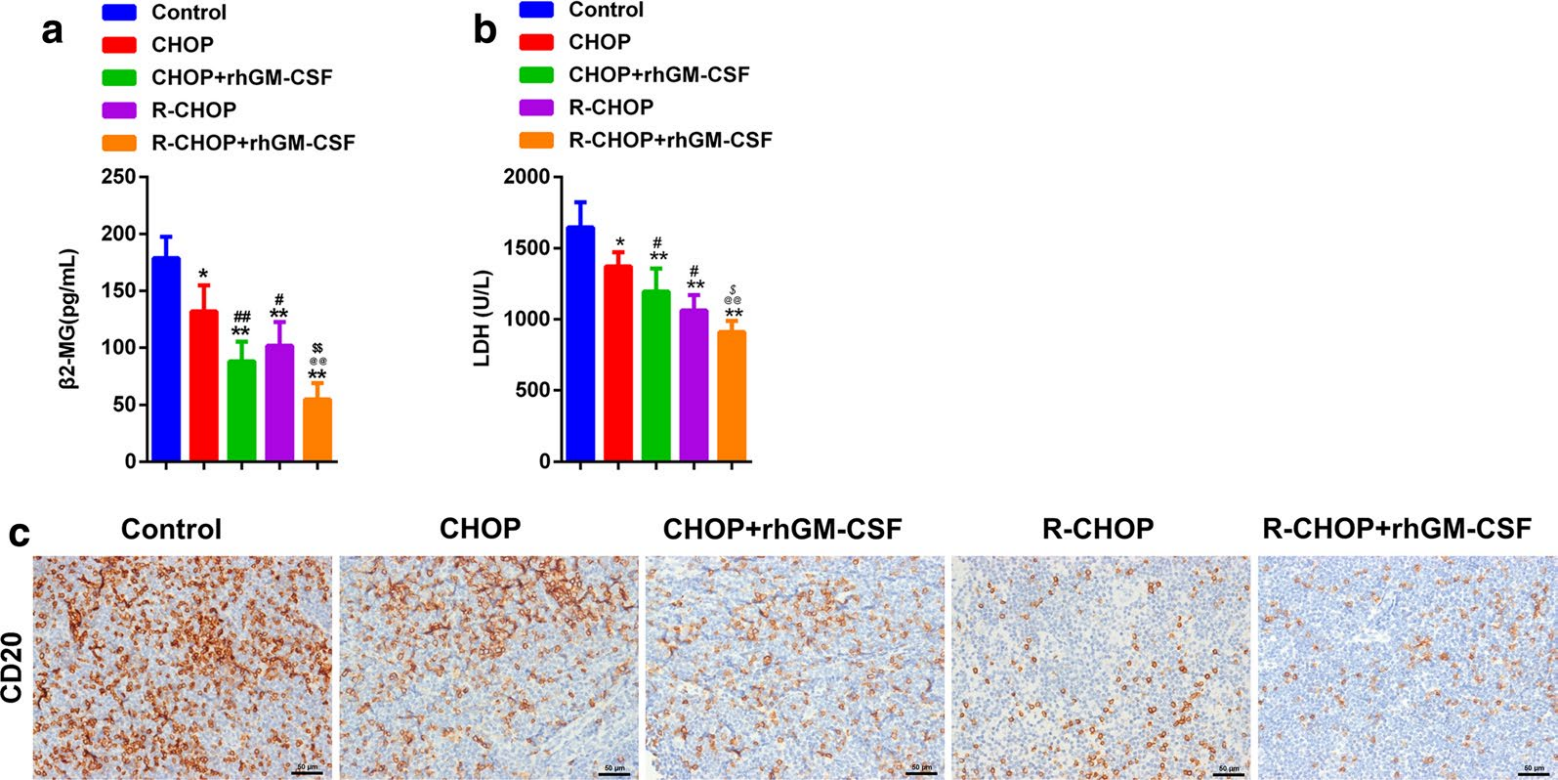
GM-CSF enhanced the effect of CHOP and R-CHOP on the production of β2-MG, LDH and CD20. **a**, **b** The ELISA assay was performed to detect the level of β2-MG, LDH in the serum of DLBCL-bearing mice. **c** IHC was performed to evaluate the expression of CD20 in the tumor of DLBCL. *p < 0.05, **p < 0.01 vs. Control, #p < 0.05 and ##p < 0.01 vs. CHOP, @@p < 0.01 vs. CHOP + GM-CSF, $p < 0.05 and $$p < 0.01 vs. R-CHOP. Data are representative of at least three independent experiments

The levels of β2-MG and LDH were closely associated with DLBCL development, so we evaluated the plasma levels of β2-MG and LDH in tumor-bearing mice. The results showed that R-CHOP regimen significantly decreased the levels of β2-MG and LDH in tumor-bearing mice. CHOP regimen showed a similar effect but affected to a lesser extent. This inhibitory effects of CHOP and R-CHOP were further amplified by GM-CSF (Fig. 2a, b). We further detected the expression of CD20, which was the target of rituximab. As expected, both CHOP and R-CHOP obviously inhibited CD20 expression in the lymphoma site of DLBCL-bearing mice, with R-CHOP exhibiting a stronger effect than CHOP. Moreover, the effects of CHOP and R-CHOP were more prominent in the presence of GM-CSF (Fig. 2c).

### GM-CSF regulated the effect of CHOP and R-CHOP on macrophage proportion in TME

**Fig. 3:**
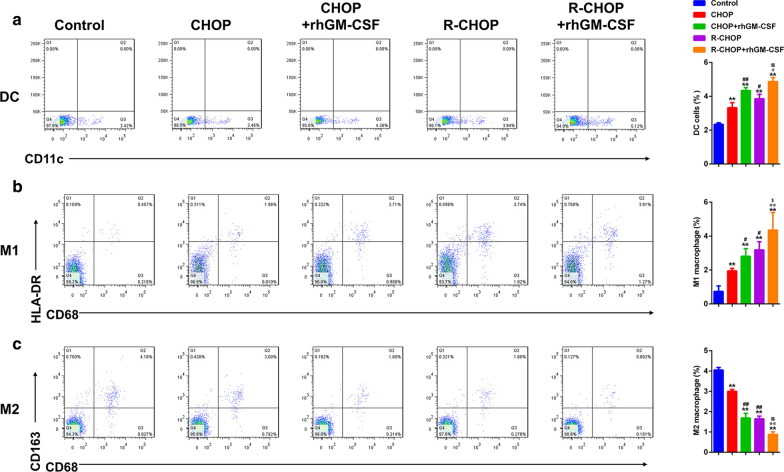
GM-CSF regulates the effect of CHOP and R-CHOP on macrophage cell proportion in TME. Flow cytometry were carried out to detect the proportion of immune cells including DC (**a**), M1 (**b**) and M2 (**c**) cells. **p < 0.01 vs. Control, #p < 0.05 and ##p < 0.01 vs. CHOP, @p < 0.05 and @@p < 0.01 vs. CHOP + GM-CSF, $p < 0.05 and $$p < 0.01 vs. R-CHOP. Data are representative of at least three independent experiments

Next, the proportions of various immune cell populations including DCs, M1 macrophages and M2 macrophages were determined in TME. As shown in Fig. 3a, both CHOP and R-CHOP treatment increased the percentages of DCs, with R-CHOP having a higher efficacy. GM-CSF administration further augmented the effect of CHOP and R-CHOP. Moreover, M1 proportion was elevated after treatment with CHOP and R-CHOP, with R-CHOP exhibiting a stronger effect than CHOP. Moreover, GM-CSF showed a synergistic effect on enhancing M1 macrophage percentage in CHOP and R-CHOP group (Fig. 3b). On the contrary, M2 proportion was reduced after CHOP and R-CHOP treatment, with R-CHOP exhibiting a stronger effect than CHOP. Moreover, the effect of CHOP and R-CHOP on M2 proportion was more prominent in the presence of GM-CSF (Fig. 3c). In summary, CHOP and R-CHOP promote DC accumulation and M1 macrophage polarization in TME, which can be further amplified by GM-CSF.

### **GM-CSF enhanced the effect of CHOP and R-CHOP on the polarization of M1 macrophages*in vitro***

**Fig. 4:**
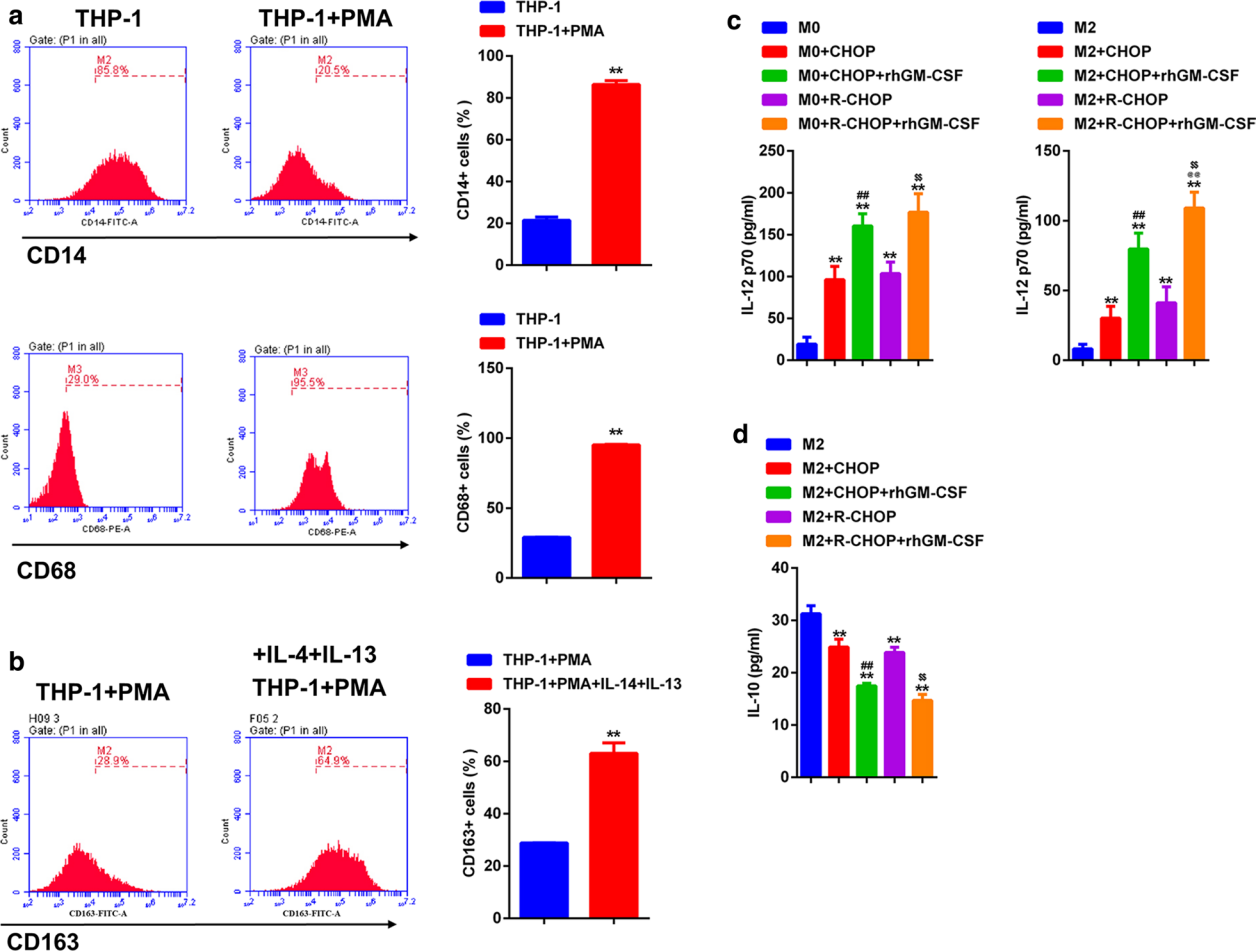
GM-CSF enhances the effect of CHOP and R-CHOP on the polarization of M1 macrophages in vitro. **a** PMA treatment induced the differentiation of THP-1 to M0 macrophages. **b** M0 macrophages were stimulated with IL-4 and IL-13, flow cytometry was carried out to detect the expression of CD163 on THP-1 cells. **c** ELISA was used to detect the IL-12 p70 level of M0 and M2 cell. **d** ELISA was used to detect the IL-10 level of M2 cells. (A)**p < 0.01 vs. THP-1, (B)**p < 0.01 vs. THP-1 + PMA, (**c** and** d**) **p < 0.01 vs. Control, ##p < 0.01 vs. CHOP, @@p < 0.01 vs. CHOP + GM-CSF, $$p < 0.01 vs. R-CHOP. Data are representative of at least three independent experiments

We then evaluated the impacts of four therapeutic regimens on macrophages polarization in vitro. To this end, PMA-induced THP-1 macrophages were used. The level of CD14 was significantly downregulated, while the level of CD68 was significantly up-regulated on THP-1 cells after PMA treatment (Fig. 4a), indicating that THP-1 cells were successfully differentiated into macrophages (M0). Thereafter, THP-1 cells were treated with IL-4 and IL-13 to induce M2 macrophages, which expressed a high level of CD163 (Fig. 4b). Then we evaluated the impact of CHOP/R-CHOP and GM-CSF on macrophage polarization. After CHOP or R-CHOP treatment, the production of IL-12 in M0 cells and M2 cells was up-regulated. The level of IL-12 was further increased by GM-CSF in both CHOP and R-CHOP groups (Fig. 4c). In contrast, the production of IL-10 in M2 cells was down-regulated by CHOP or R-CHOP. The downregulation of IL-10 was more prominent in the presence of GM-CSF (Fig. 4d). Therefore, CHOP/R-CHOP treatment promotes the polarization of M1 macrophages, which can be further amplified by GM-CSF.

### GM-CSF enhanced the anti-DCBCL effect of CHOP and R-CHOP-challenged macrophages

**Fig. 5:**
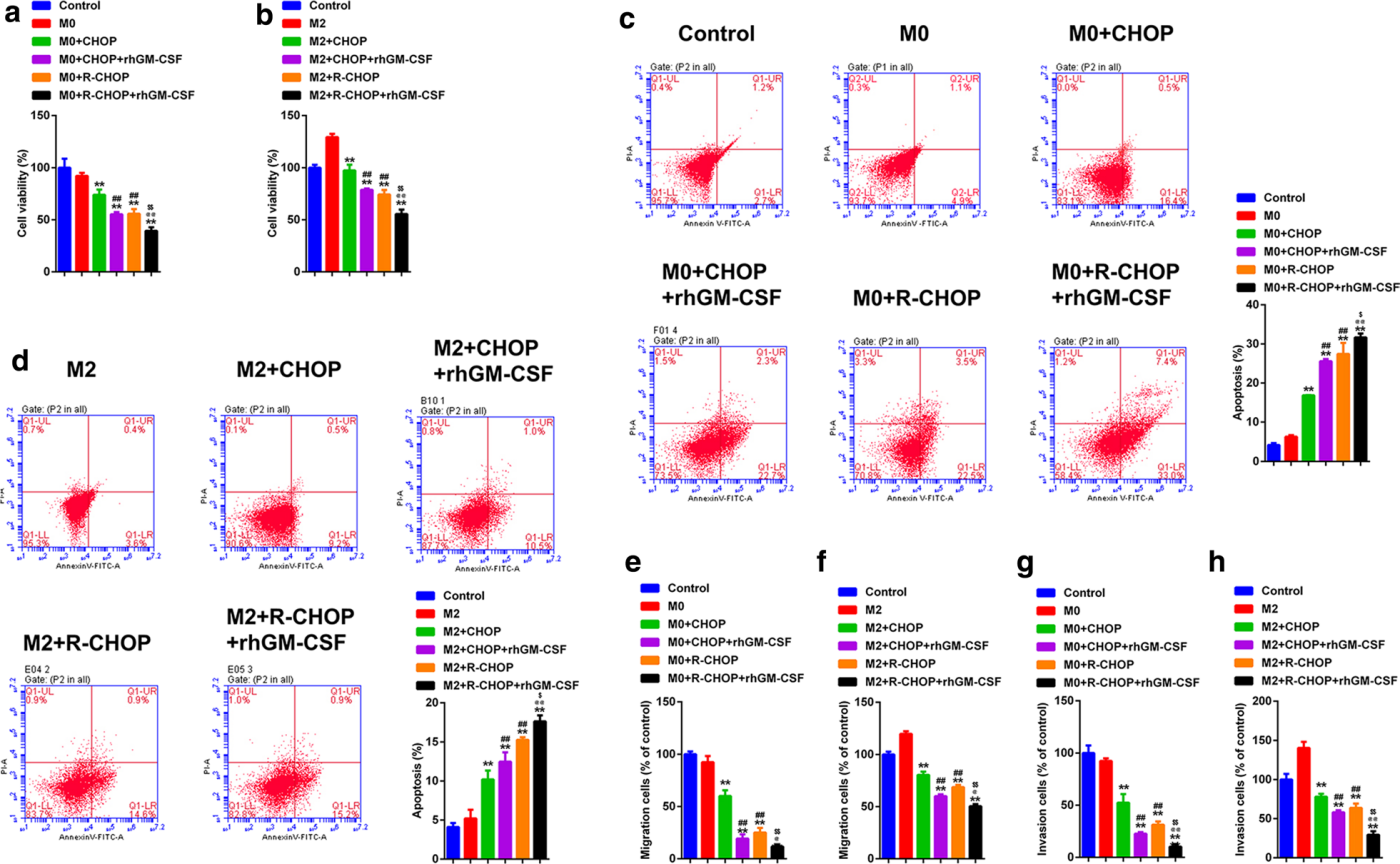
GM-CSF enhanced CHOP and R-CHOP to promote the anti-DCBCL effect of macrophages. **a**, **b** CCK-8 was used to assess the cell proliferation of DLBCL cells. **c**, **d** Flow cytometry and Annexin V-FITC/PI staining was used to evaluate the cell apoptosis of DLBCL cells. Transwell was performed to detect the migration **e**, **f** and invasion (**g**,** h**) of DLBCL cells. **p < 0.01 vs. M0/M2, ##p < 0.01 vs. M0/M2 + CHOP, @@p < 0.01 vs. M0/M2 + CHOP + GM-CSF, $p < 0.05 and $$p < 0.01 vs. M0/M2 + R-CHOP. Data are representative of at least three independent experiments

We asked if the four therapeutic regimens for DLBCL could influence tumor cell behaviors. To this end, M0 and M2 macrophages were pre-treated with these regimens, then co-cultured with SU-DHL-4 cells. The results showed that, compared to control macrophages, CHOP and R-CHOP-pretreated M0 and M2 macrophages reduced the proliferation, migration, and invasion of SU-DHL-4 cells. In contrast, pre-treated macrophages induced apoptosis of SU-DHL-4 cell. All these effects were more obvious in R-CHOP group than those in CHOP group. Moreover, compared to CHOP and R-CHOP treatment alone, GM-CSF enhanced anti-tumor capacity of CHOP and R-CHOP-treated macrophages (Fig. 5a–h). Taken together, the CHOP and R-CHOP therapeutic regimens facilitate the anti-tumor phenotype switch of macrophages, which is further augmented by GM-CSF.

### AKT signaling was required in GM-CSF-augmented the anti-DCBCL effect of CHOP and R-CHOP

**Fig. 6:**
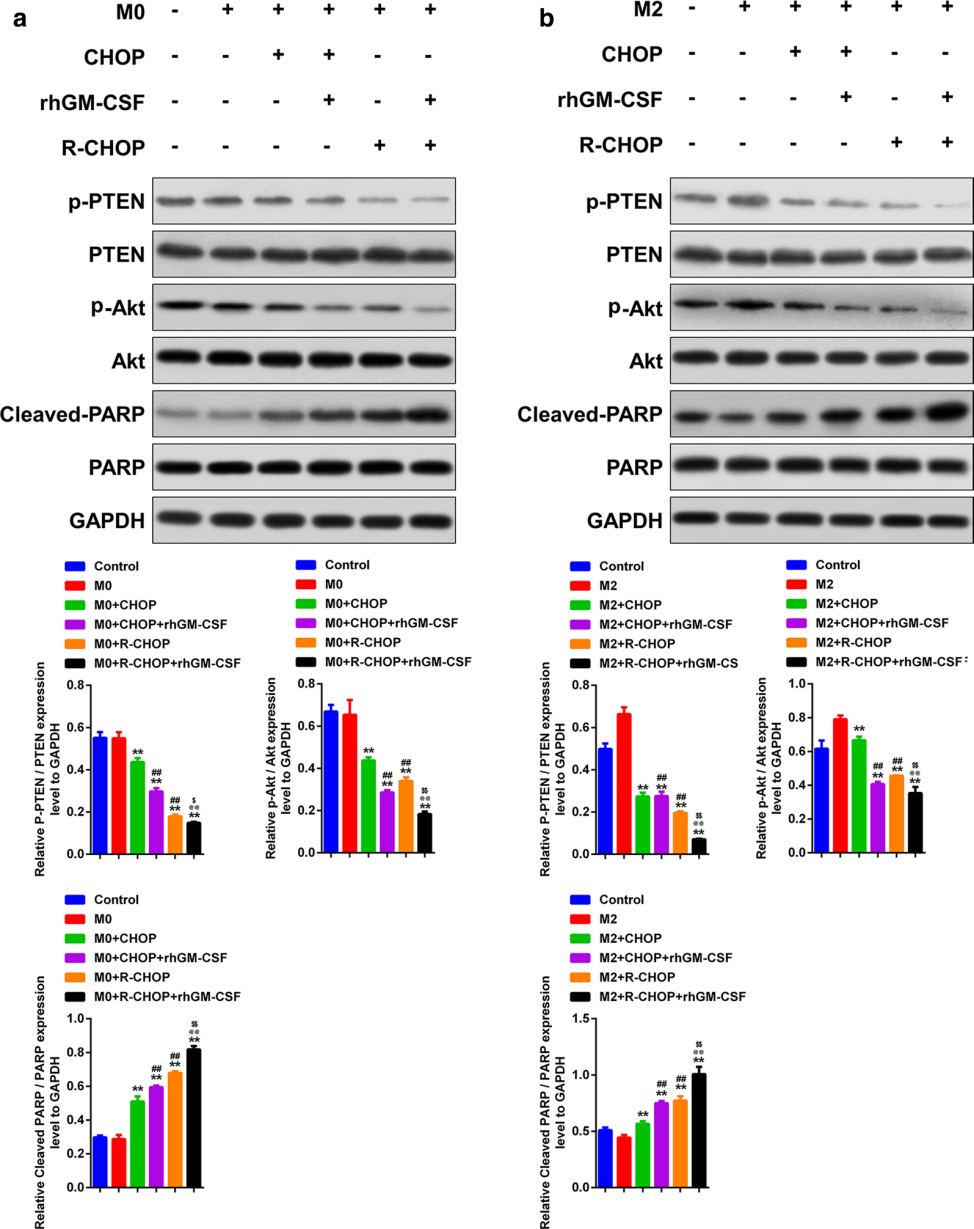
GM-CSF enhanced CHOP and R-CHOP to promote the activation of AKT signaling in DLBCL cells by macrophages. **a**, **b** Western blot was used to detect the phosphorylation of AKT and PTEN, and the expression of cleaved PARP. **p < 0.01 vs. M0/M2, ##p < 0.01 vs. M0/M2 + CHOP, and @@p < 0.01 vs. M0/M2 + CHOP + GM-CSF, $p < 0.05 and $$p < 0.01 vs. M0/M2 + R-CHOP.Data are representative of at least three independent experiments

Finally, western blot was carried out to evaluate the differences of signaling transduction in SU-DHL-4 cells co-cultured with macrophages. As shown in Fig. 6, the phosphorylation of AKT and PTEN in SU-DHL-4 cells was dampened when co-cultured with CHOP and R-CHOP treated M0 or M2 macrophages. In addition, the level of cleaved-PARP, a marker of cell apoptosis, was elevated in SU-DHL-4 cells co-cultured with CHOP and R-CHOP-primed macrophages, with R-CHOP possessing a higher efficacy. Importantly, GM-CSF acted synergistically with CHOP and R-CHOP to inhibit AKT and PTEN phosphorylation and to enhance PARP cleavage (Fig. 6).

## Discussion

In the current clinical applications for DLBCL, the standard therapeutic program CHOP and R-CHOP significantly improve the overall survival of patients with DLBCL. However, approximately 30 % DLBCL patients display chemo-resistance and are irresponsive to CHOP and R-CHOP therapy. Possibly, a better understanding of the mechanisms by which CHOP/R-CHOP and GM-CSF-mediated the beneficial functions, especially their roles in reshaping the tumor microenvironment, is of great importance for the further optimization of CHOP-based therapeutics.

In the present study, we compared the effects of CHOP and R-CHOP on DLBCL development. Consistent with a previous report, R-CHOP has a better anti-tumor efficacy than CHOP [17]. The effects are due to the capacity of rituximab to enhance CD20 antigen expression, and subsequently augment antibody-dependent cytotoxicity and immune cell proliferation [18]. Moreover, we then tested the potential synergistic effect of GM-CSF on CHOP and R-CHOP regimens, because GM-CSF has been reported to boost anti-tumor immunity and improve prognosis in patients receiving chemotherapy [19]. The results showed that GM-CSF acted synergistic effects with CHOP/R-CHOP to suppress DLBCL development, suggesting that GM-CSF plus CHOP/R-CHOP might be a promising combination therapeutics.

Tumor microenvironment (TME) has been well-accepted to play crucial roles in cancer progression [20]. It is reported that the cyclophosphamide/doxorubicin/vincristine combination treatment facilitates the repolarization of macrophages into an anti-tumor phenotype in a B16 melanoma model [21]. Moreover, macrophages have been reported to greatly affect the outcome of CHOP and R-CHOP chemotherapy in DLBCL [22, 23], suggesting that CHOP/R-CHOP may play a role in shaping the functions of tumor-associated macrophages in DLBCL. Nevertheless, how CHOP and R-CHOP influence macrophage polarization in TME of DLBCL is still elusive. Our work uncovered that CHOP and R-CHOP treatment increased the proportions of DCs and M1 macrophages, and decreased the proportion of M2 macrophages in DLBCL-bearing mice. The *in vitro* study indicated that CHOP and R-CHOP reduced the functions of pro-tumoral M2 macrophages, and enhanced the functions of anti-tumoral M1 macrophages, suggesting CHOP and R-CHOP therapeutic regimen could boost macrophage-mediated anti-tumor immune responses. On the other hand, although we found that R-CHOP had higher efficacy in promoting M1 polarization while reducing M2 polarization than CHOP in DLBCL-bearing mice, this effect was not observed in the *in vitro* experiment in terms of IL-10 and IL-12 production. It is likely that rituximab inhibits the growth of DLBCL cells by targeting CD20 in tumor microenvironment, thereby preventing the polarization of M2 macrophages indirectly. Possibly, rituximab fails to regulate macrophage polarization *in vitro* due to the absence of DLBCL cells, which is consistent with a report showing that rituximab alone has negligible impact on macrophage polarization [24]. Importantly, we found that GM-CSF had an obvious synergistic effect with CHOP/R-CHOP on enhancing M1 polarization from both M2 macrophages and M0 macrophages, indicating the dual role of GM-CSF in re-educating M2 macrophages in TME and newly-differentiated peripheral monocytes. However, due to the lack of adaptive immune system in nude mice, the influences of CHOP regimens on tumor-infiltrating CD4 + cells and CD8 + T cells, as well as T cell-derived cytokines were not investigated in our present work. Further efforts are needed to explore this issue using DLBCL cells in immunocompetent mice.

It is well-accepted that macrophages contribute to tumor progression by promoting the proliferation, migration and invasion of cancer cells through multiple mechanisms [15, 25]. For example, macrophage-derived exosomes can be ingested by gastric cancer cells, reducing their chemotherapy sensitivity to cisplatin [26, 27]. Moreover, M2-polarized tumor-associated macrophages is demonstrated to be capable of promoting EMT through AKT3 activation in intrahepatic cholangiocarcinoma [15]. Here, we firstly indicated that CHOP-challenged macrophages reduced several malignant behaviors of DLBCL cells, including proliferation, migration, and invasion. The synergistic roles of rituximab and GM-CSF were also reported. CHOP regimen exerts inhibitory effects on tumor behaviors of DLBCL via the activation of AKT signaling, a well-characterized oncogenic pathway in most cancer cells. Similarly, in this study, the negative role of CHOP on AKT activation was further augmented by rituximab and GM-CSF. However, the specific macrophage-derived mediators that are responsible for inhibiting AKT signaling in DLBCL cells still need further identification. Additionally, whether GM-CSF enhances the sensitivity tumor cells to CHOP/R-CHOP shall be explored by further study. Moreover, loss of CD20 antigen may induce the insensitivity to rituximab in recurrent DLBCL. The role of GM-CSF on the sensitivity of lymphoma cells to rituximab also needs the future research.

## Conclusions

In conclusion, here we reported that the CHOP and R-CHOP facilitated the polarization of anti-tumor macrophages in DLBCL immune microenvironment for the first time. GM-CSF exhibited promising synergistic functions in improving the therapeutic efficacy of CHOP and R-CHOP. Our findings highlight the crucial roles of CHOP and R-CHOP, either using alone or in combination with GM-CSF, on the re-polarization of tumor-associated macrophages in DLBCL, thus providing an optimizing strategy for CHOP-based DLBCL treatment. However, more detailed molecular mechanisms are needed to explore further, clarifying how CHOP and R-CHOP influences the functions of macrophages, and how GM-CSF acts synergistically with CHOP and R-CHOP regimen.

## Data Availability

All data generated or analyzed during this study are included in this published article.

## Abbreviations

DLBCL: Diffuse large B-cell lymphoma
NHLs: Non-Hodgkin lymphomas
CHOP: Cyclophosphamide/doxorubicin/vincristine/prednisone
M1: Macrophages
M2: Macrophages
